# Steep‐Slope Gate‐Connected Atomic Threshold Switching Field‐Effect Transistor with MoS_2_ Channel and Its Application to Infrared Detectable Phototransistors

**DOI:** 10.1002/advs.202100208

**Published:** 2021-05-03

**Authors:** Seung‐Geun Kim, Seung‐Hwan Kim, Gwang‐Sik Kim, Hyeok Jeon, Taehyun Kim, Hyun‐Yong Yu

**Affiliations:** ^1^ Department of Semiconductor Systems Engineering Korea University 145, Anam‐ro, Seongbuk‐gu Seoul 02841 Korea; ^2^ School of Electrical Engineering Korea University 145, Anam‐ro, Seongbuk‐gu Seoul 02841 Korea

**Keywords:** atomic threshold switching field‐effect transistors, high detectivity, infrared detectable phototransistors, low subthreshold swing, 2D channel

## Abstract

For next‐generation electronics and optoelectronics, 2D‐layered nanomaterial‐based field effect transistors (FETs) have garnered attention as promising candidates owing to their remarkable properties. However, their subthreshold swings (*SS*) cannot be lower than 60 mV/decade owing to the limitation of the thermionic carrier injection mechanism, and it remains a major challenge in 2D‐layered nanomaterial‐based transistors. Here, a gate‐connected MoS_2_ atomic threshold switching FET using a nitrogen‐doped HfO_2_‐based threshold switching (TS) device is developed. The proposed device achieves an extremely low *SS* of 11 mV/decade and a high on‐off ratio of ≈10^6^ by maintaining a high on‐state drive current due to the steep switching of the TS device at the gate region. In particular, the proposed device can function as an infrared detectable phototransistor with excellent optical properties. The proposed device is expected to pave the way for the development of future 2D channel‐based electrical and optical transistors.

## Graphical abstract

Gate‐connected atomic threshold switching field‐effect transistor with MoS_2_ channel using threshold switch mechanism is investigated to achieve low subthreshold swing of 11.1 mV/decade for various electric and optoelectronic nanodevices. In addition, the proposed device can be used as an infrared detectable phototransistor and achieves significantly high optical performance (*I*_light/dark_ = 4.7 × 10^4^ and *D* ^*^ = 2.7 × 10^12^ cmHz^0.5^W^−1^).

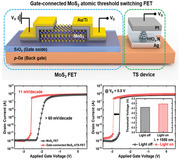

## 1 Introduction

2D‐layered nanomaterials, such as graphene,^[^ ^1^, ^2^, ^3^, ^4^, ^5^, ^6^, ^7^ ^]^ black phosphorus,^[^ ^8^, ^9^, ^10^, ^11^, ^12^, ^13^ ^]^ and transition metal dichalcogenides (TMDCs),^[^ ^14^, ^15^, ^16^, ^17^, ^18^, ^19^, ^20^, ^21^, ^22^, ^23^ ^]^ have received substantial attention in the fields of nanoelectronics and optoelectronics in the past few years. Recent studies on devices fabricated using 2D material for channel have revealed that they have excellent current on/off ratio, satisfactory charge carrier mobility, low subthreshold swing (*SS*), and interesting optical properties.^[^ ^1^, ^2^, ^3^, ^4^, ^5^, ^6^, ^7^, ^8^, ^9^, ^10^, ^11^, ^12^, ^13^, ^14^, ^15^, ^16^, ^17^, ^18^, ^19^, ^20^, ^21^, ^22^, ^23^ ^]^ Although conventional 2D‐channel‐based field‐effect transistors (FETs) exhibit a low *SS* value, the *SS* cannot be lower than 60 mV/decade at room temperature owing to the limitation of the thermionic carrier injection mechanism.^[^ ^24^, ^25^, ^26^ ^]^ The *SS* of the transistor needs to be as low as possible to maintain the total power consumption, and a low *SS* leads to a high switching speed and low power consumption per device.^[^ ^26^, ^27^ ^]^ To overcome the *SS* limit, several types of steep‐slope 2D‐channel devices have been proposed such as tunneling FET,^[^ ^13^, ^28^, ^29^, ^30^ ^]^ negative capacitance FET,^[^ ^24^, ^25^, ^31^, ^32^ ^]^ and phase FET.^[^ ^26^, ^33^, ^34^ ^]^

In particular, phase FETs are currently considered the most promising steep‐slope device. The concept of phase FET was first implemented by Shukla et al. in 2015 using an insulator‐to‐metal transition (IMT) material, VO_2_, integrated in series with the source of the conventional FET.^[^ ^35^ ^]^ When external perturbations such as temperature, pressure, and electrical stimulus are applied to VO_2_, phase transition is induced, which causes an abrupt change in conductivity. The phase FET using VO_2_ shows excellent properties such as an abrupt increase in current with applied voltage, short switching time, high on‐current, low fabrication temperature, and high compatibility with conventional complementary metal‐oxide‐semiconductor technology. Owing to these advantages, in 2017, Grisafe et al. applied the phase FET concept with VO_2_ in the MoS_2_‐channel FET to further boost the 2D‐channel device performance.^[^ ^33^ ^]^ The suggested MoS_2_‐channel phase FET has a low operation voltage and low *SS*, but it involves several problems, such as a high off‐state leakage current and low thermal stability, according to the limitations of the IMT materials. In 2019, HfO_2_‐based threshold switching (TS) device was suggested for MoS_2_‐channel phase FETs instead of IMT materials, because of the low off‐state leakage current characteristic of ≈1 pA and the superior thermal stability of ≈90 °C.^[^ ^34^ ^]^ However, these source‐connected phase FETs decrease not only the off‐state leakage current but also the on‐state drive current, regardless of the material used in the TS device because the TS device is located in the main current flow path. Therefore, research on realizing steep switching devices while maintaining the superior characteristics of MoS_2_ FET needs to be conducted.

Achieving a low *SS* value of FETs in terms of the optical concept is also important because one of the main challenges in optics is reducing the total power consumption in optoelectronics.^[^ ^36^ ^]^ The *SS* corresponds to the gating efficiency, such that reducing the *SS* makes it possible to ensure low voltage operability.^[^ ^37^ ^]^ In addition, the photogating effect‐based phototransistor, which operates the photogenerated carriers similar to the back‐gate voltage in a FET, has a close relationship with gating efficiency because the optical operating mechanism is threshold voltage shifting.^[^ ^38^ ^]^ Therefore, the improvement in gating efficiency by decreasing the *SS* can enhance the optical characteristics of photogating effect‐based phototransistors. However, few studies have focused on lowering the *SS* value of phototransistors, and research on this aspect is essential for next‐generation optoelectronics.

Here, we demonstrate a gate‐connected MoS_2_ atomic threshold switching FET (ATS‐FET) using a nitrogen‐doped HfO_2_ (HfO*_x_*:N)‐based TS device for a next‐generation steep‐slope 2D‐channel device. The on‐state drive current of a MoS_2_ ATS‐FET is maintained owing to the gate‐connected structure and the *SS* of the device is reduced through the Ag conductive filament formed and ruptured in the HfO*_x_*:N layer. In particular, the proposed device can detect the infrared range light owing to the special properties of the Ge substrate. Therefore, the proposed ATS‐FET can also be used as an infrared detectable phototransistor based on the photogating effect. Compared with the conventional MoS_2_ phototransistor, the optical characteristics of the proposed device are enhanced by the combined action of the photogating effect and the low *SS*. It was also successfully confirmed that the responsivity and detectivity for the incident infrared light were increased.

## 2 Results and Discussion

### 2.1 Device Scheme and Typical Characteristics

**Figure 1:**
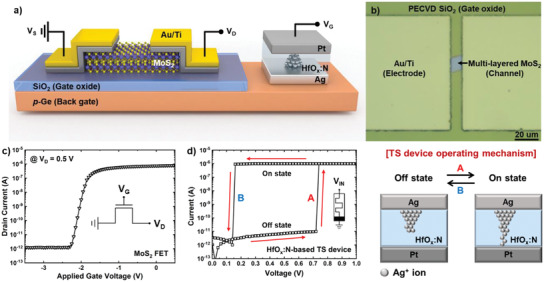
Characterizations of gate‐connected MoS_2_ ATS‐FET. a) Schematic of gate‐ connected MoS_2_ ATS‐FET. b) Optical image of the multi‐layer MoS_2_ device with Au/Ti as the source and drain electrodes. c) *I*_D_–*V*_G_ characteristics of the MoS_2_ FET. d) Typical *I*–*V* characteristic and schematic of the HfO*_x_*:N‐based TS device to explain the operating mechanism.

A schematic of the gate‐connected MoS_2_ ATS‐FET is shown in **Figure** **1**a. The proposed ATS‐FET was formed by connecting the MoS_2_ FET gate electrode in series with the HfO*_x_*:N‐based TS device. First, the MoS_2_ FET was fabricated using *p*‐type Ge (*p*‐Ge) (*N*_a_ = 1 × 10^16^ cm^−3^), which is used for the back‐side gate electrode. A 50‐nm‐thick silicon dioxide (SiO_2_) layer was deposited on the *p*‐Ge substrate via plasma‐enhanced chemical vapor deposition to form a gate oxide. Then, the MoS_2_ flake as a channel was transferred onto the SiO_2_/*p*‐Ge substrate using the polydimethylsiloxane‐based mechanical exfoliation method. A 40‐nm‐thick Ti layer was deposited as a source/drain (S/D) contact metal. The S/D contact metal was fabricated in parallel with a spacing of 10 µm, as shown in the top‐view optical microscope image of Figure 1b. As shown in Figure S1, Supporting Information, two conventional peaks (E^1^_2g_ and A_1g_) of the MoS_2_ were clearly revealed at 382.2 and 407.6 cm^−1^ by Raman spectroscopy, indicating that the MoS_2_ is present as a multi‐layer.^[^ ^39^ ^]^ The thickness of the MoS_2_ was measured to be 11 nm by using atomic force microscopy (AFM), as shown in Figure S2, Supporting Information, which indicates that MoS_2_ has ≈15 layers. Figure 1c shows the drain current–gate voltage (*I*_D_–*V*_G_) characteristics of the MoS_2_ FET, which exhibited n‐type transfer characteristics with a high on‐state current of ≈10^−6^ A and a high current on/off ratio of ≈10^6^, similar to typical multi‐layered MoS_2_ FETs.^[^ ^40^ ^]^

The HfO*_x_*:N‐based TS device with an Ag/HfO*_x_*:N/Pt/Ti structure, which is essential for constructing the ATS‐FET, was fabricated on a 90‐nm‐thick SiO_2_/Si substrate. Ag (100 nm), Pt (15 nm), and Ti (10 nm) metal layers were deposited via electron‐beam evaporation. A 50‐nm‐thick HfO*_x_*:N interlayer was deposited using radio frequency sputtering with a HfO_2_ ceramic target under a N_2_ gas flow of 2 sccm. Through the top‐view optical microscope image and cross‐sectional view transmission electron microscopy image, the TS device was confirmed that the structure of that was completed, as shown in Figure S3, Supporting Information. Figure 1d shows the typical *I*–*V* characteristics and the operation mechanism of the HfO*_x_*:N‐based TS device under the direct current sweep. The TS device shows an abrupt resistance switching during forward and backward voltage sweeping, which can be explained by the formation and dissolution of the Ag filament in the HfO_2_ electrolyte.^[^ ^41^, ^42^, ^43^, ^44^ ^]^ When applying a forward sweep from 0 to 1 V, an Ag filament is formed between the two electrodes and the TS device changes from the off‐state to the on‐state. In contrast, when applying a backward sweep from 1 to 0 V, the device is turned off because the Ag filament is spontaneously ruptured to minimize the interfacial energy.

### 2.2 Electrical Properties of Gate‐Connected MoS_2_ ATS‐FET

**Figure 2:**
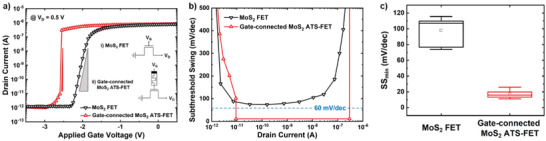
Electrical characterization of gate‐connected MoS_2_ ATS‐FET. a) *I*_D_–*V*_G_ characteristics of the gate‐connected MoS_2_ ATS‐FET. The inset shows circuit schematics of FETs with and without the TS device in series with the gate of a conventional MoS_2_ FET. b) Subthreshold swing as a function of *I*_D_ of the gate‐connected MoS_2_ ATS‐FET. c) Distribution of *SS*_min_ in 10 devices.

**Figure** **2** shows the electrical properties of gate‐connected MoS_2_ ATS‐FET, which was formed by connecting the MoS_2_ FET gate electrode in series with the HfO*_x_*:N‐based TS device. Figure 2a shows the *I*_D_–*V*_G_ characteristics of the MoS_2_ FETs with and without the TS device in the gate region at *V*_G_ from 0.5 to −3.5 V with a step of 0.05 V with constant *V*_D_ = 0.5 V. The threshold voltage (*V*_TH_) of the gate‐connected MoS_2_ ATS‐FET is shifted toward the left side as compared with the MoS_2_ FET from −2.19 to −2.60 V (the *V*_TH_ was defined for *V*_G_ when *I*_D_ = 10^−11^ A). The higher |*V*_TH_| of the gate‐connected MoS_2_ ATS‐FET indicates that the applied gate voltage is not used entirely to turn off the transistor. In the case of drain current for both devices, the on‐ and off‐state currents are ≈10^−6^ and 10^−12^ A, respectively, and the on/off switching ratio is ≈10^6^. The reason for the same on‐ and off‐state currents in both devices is that the TS device is connected to the gate rather than the channel through which current flows directly, and therefore it does not interfere with the flow of the drive current. In addition, a sharp rise in the drain current is observed while maintaining the on‐ and off‐state current values. This change in drain current results in a dramatic decrease in the minimum *SS* value, as shown in Figure 2b. The *SS*s of the two devices are obtained from the transfer characteristics at *V*_D_ = 0.5 V, where $SS\;=\frac{\partial V_{G}}{\partial (logI_{D})}\;$. While the MoS_2_ FET had a minimum *SS* of 73.8 mV/decade, the gate‐connected MoS_2_ ATS‐FET achieved a significantly low minimum *SS* (*SS*_min_ *SS*) of 11.1 mV/decade with a range of abrupt current transition close to 10^4^. Besides, Figure 2c shows the distribution of *SS*_min_ *SS* in 10 devices to confirm the device‐to‐device variation. As a result, the value and variation of *SS*_min_ *SS* of ATS‐FET tend to decrease compared to that of MoS_2_ FET and it shows that our proposed device has good reliability. The *I*_D_–*V*_G_ characteristics and *SS*_min_ *SS* values for various *V*_G_ steps are shown in Figure S4, Supporting Information. It was confirmed that as the *V*_G_ step became finer, more data points appeared on the *SS* slope, and the *SS*_min_ *SS* values are maintained at a very low value compared to the conventional MoS_2_ FET. Furthermore, as a result of the *I*_D_–*V*_D_ measurement (Figure S5, Supporting Information), it was confirmed that a large gap was formed between the drain current values according to the gate step due to the low *SS* of the ATS‐FET.

**Figure 3:**
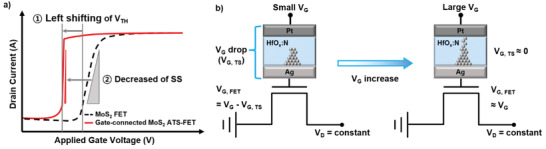
Mechanism of steep‐switching in gate‐connected MoS_2_ ATS‐FET. a) Schematic of transfer curves of the MoS_2_ FET and gate‐connected MoS_2_ ATS‐FET showing changes of *V*_TH_ and *SS*. b) Circuit illustrations of gate‐connected MoS_2_ ATS‐FET at small and large *V*_G_.

To explain the modulation of the electrical properties of the gate‐connected MoS_2_ ATS‐FET, the schematic *I*_D_–*V*_G_ curves and circuits are illustrated in **Figure** **3**. Figure 3a shows a schematic illustration of the transfer *I*_D_–*V*_G_ curve with and without the TS device at the gate region. As shown in Figure 3a, the gate‐connected MoS_2_ ATS‐FET shows a left shift of *V*_TH_ and a decrease of *SS* compared to the MoS_2_ FET, when sweep *V*_G_ is in the negative direction. These modulations can be explained by the equivalent circuit and voltage distribution for the device, as shown in Figure 3b. When low *V*_G_ is applied, most of the voltage drops occur on the TS device owing to the high resistance, as shown on the left side of Figure 3b. Therefore, the applied gate voltage of the MoS_2_ FET (*V*_G,FET_) remains low and the MoS_2_ channel remains in the on‐state. For this reason, *V*_TH_ of the gate‐connected MoS_2_ ATS‐FET was shifted toward the left side. As *V*_G_ increases in the negative direction, the voltage applied to the TS device (*V*_G,TS_) increases, eventually forming an Ag filament between the two electrodes to turn on the TS device, as shown on the right side of Figure 3b. Then, the TS device changes to a low resistance state, which is makes the extremely low *V*_G,TS_ drops, and *V*_G,FET_ changes steeply to a value approaching *V*_G_. As a result of the sudden change in *V*_G,FET_, the *SS* of the gate‐connected MoS_2_ ATS‐FET decreases significantly as compared with that of the MoS_2_ FET. According to the above‐mentioned, the main factor determining *V*_TH_ and *SS* of the gate‐connected MoS_2_ ATS‐FET is the formation and dissolution of the filament of the TS device in the gate region.

### 2.3 Optical Properties of Gate‐Connected MoS_2_ ATS‐FET

**Figure 4:**
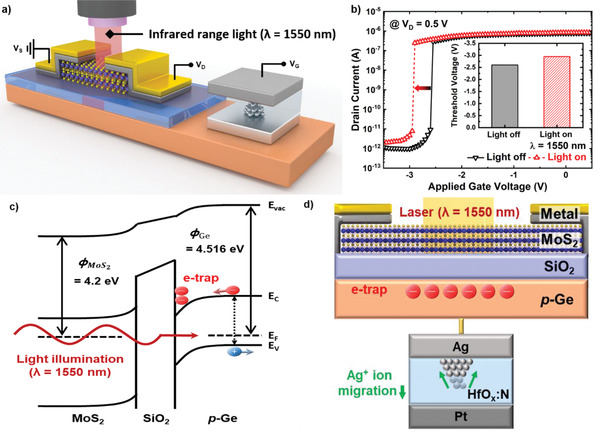
Optical characterization and operating mechanism of gate‐connected MoS_2_ ATS‐FET. a) Schematic of the gate‐connected MoS_2_ ATS‐FET under incident light (*λ* = 1550 nm). b) *I*_D_–*V*_G_ characteristics of the gate‐connected MoS_2_ ATS‐FET with and without 1550 nm infrared light. The inset shows threshold voltages with and without the incident light. c) Energy band diagram of the MoS_2_ FET in the vertical direction under infrared light using a Ge gate. d) Cross‐section illustration of the gate‐connected MoS_2_ ATS‐FET under incident light, indicting a reduction in migration of Ag^+^ ions.

In general, MoS_2_ FETs have been widely studied as optoelectronic devices.^[^ ^16^, ^18^, ^20^, ^40^, ^45^ ^]^ Similarly, the proposed gate‐connected MoS_2_ ATS‐FET is also capable of photodetection. To assess the photodetection performance, the infrared light (*λ* = 1550 nm) was incident perpendicularly and uniformly onto the channel region of the device shown in **Figure** **4**a. The *I*_D_–*V*_G_ characteristics of the gate‐connected MoS_2_ ATS‐FET with and without irradiation of 1550 nm infrared light are presented in Figure 4b. With irradiation, the ATS‐FET exhibited a negative *V*_TH_ shift from −2.60 to −2.95 V (inset in Figure 4b) while maintaining a low *SS* of 11.1 mV/decade. In addition, rise time of 51.1 ms and decay time of 27.3 ms were measured as shown in Figure S6, Supporting Information, and the rise and decay time values were extracted between 10% and 90% of the increasing and decreasing drain current. The principle of threshold shift under incident infrared light can be explained using a band diagram and a schematic of the proposed device as shown in Figure 4c,d. Figure 4c shows a band diagram of the channel/gate oxide/gate electrode of the MoS_2_ FET. Under thermal equilibrium conditions, initial band bending is caused by the difference in work functions between MoS_2_ ($\phi_{\mathrm{Mo}S_{2}}=\;4.2\;\mathrm{eV}$) and Ge (*φ*_Ge_ =  4.516 eV) as shown in the band diagram. When infrared light is incident onto the device, the MoS_2_ and SiO_2_ layers hardly absorb the light and act as transparent windows owing to their wide bandgaps. Therefore, the incident light can reach Ge and is absorbed owing to the narrow bandgap of Ge (*E*_g_ = 0.66 eV). The absorbed light creates electron–hole pairs in the Ge region, and the generated electrons accumulate at the SiO_2_/Ge interface because of the initial band bending. The accumulated electrons can cause two phenomena. One is decrease of the n‐type doping in the MoS_2_ channel, and the other is inhibition of the Ag^+^ ion migration in the HfO*_x_*:N layer, as shown in Figure 4d. Decrease of the n‐type doping in the MoS_2_ channel causes right shifting of the transfer curve, and inhibition of the Ag^+^ ion migration causes left shifting of the transfer curve. In the case of the gate‐connected MoS_2_ ATS‐FET, the switch operation is determined by the turn on and off of the TS device. Therefore, the transfer curve is shifted to the left side, as shown in Figure 4b. This phenomenon, in which the threshold voltage is shifted through the charge generated by incident light, is called the photogating effect.^[^ ^38^, ^46^, ^47^ ^]^

**Figure 5:**
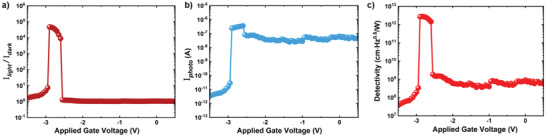
Calculated optical properties of gate‐connected MoS_2_ ATS‐FET. a) Light to dark current ratio (*I*_light_/*I*_dark_), b) photocurrent (*I*_photo_ = *I*_light_  − *I*_dark_), and c) detectivity ( D* =  *R*(*A*_device_/2*eI*_dark_)^0.5^) as a function of *V*_G_.

To further evaluate the performance of the phototransistor, we extract the light to dark current ratio, photocurrent, responsivity, external quantum efficiency (EQE), and detectivity from the *I*_D_–*V*_G_ curves. As shown in **Figure** **5**a, an ultrahigh light‐to‐dark current ratio can be obtained in the threshold shift region because the threshold shift and the significantly steep *SS* operate simultaneously in the proposed device, and the maximum value of the factor is 4.7 × 10^4^ at *V*_G_ = −2.9 V and *V*_D_ = 0.5 V. The photocurrent ( *I*_photo_ = *I*_light_  − *I*_dark_) shows a rapid change based on threshold voltage due to the threshold shift according to the incident light, and maximum values as high as 3.58 × 10^−7^ A are achieved as shown in Figure 5b. Because the responsivity (*R*) and the EQE, as shown in Figure S7a and S7b, Supporting Information, are parameters related to *I*_photo_, they exhibit the same trend of change as *I*_photo_. *R* indicates the ratio of the generated photocurrent and effective incident optical power (*R*  = *I*_photo_/*P*_eff_), and *P*_eff_ is calculated as *P*_eff_ =  *P*(*A*_device_/*A*_spot_), where *P* is the actual output laser power and *A* is the area of the device and laser spot, and the value of *P*_eff_ is 1.69 × 10^−7^ W. EQE is the ratio of the number of photoexcited charge carriers to the number of incident photons on the device from the outside; it can be expressed as EQE  =  h*cR*/*λe*, where h is Planck constant, *c* is the speed of light, *λ* is the wavelength of incident light, and *e* is the electron charge. *R* and EQE increased steeply at the subthreshold region and reached the highest values of 2.1 AW^−1^ and 169.0%, respectively. Detectivity ( D* =  *R*(*A*_device_/2*eI*_dark_)^0.5^), one of the most important parameters for photodetectors, is defined as the signal‐to‐noise ratio of the photodetector normalized by the device area. Because of the ultrahigh light‐to‐dark current ratio in the threshold shift region, D* was measured to have a considerably high value of 2.7 × 10^12^ cmHz^0.5^W^−1^ as shown in Figure 5c. All parameters mentioned above have incomparable selectivity for the applied gate voltage owing to the extremely low *SS* and threshold shift. The optical performance of the gate‐connected MoS_2_ ATS‐FET is dramatically enhanced, as compared with that of the conventional MoS_2_ FET (Figure S8, Supporting Information), which is without the TS device. A comparison of the critical optical performance indicators is presented in Table S1, Supporting Information. These optical characteristics prove that the ultra‐low *SS* of the gate‐connected MoS_2_ ATS‐FET can be beneficial when a gate‐connected MoS_2_ ATS‐FET is used as a photodetector. Compared to recently reported advanced phototransistors, as shown in **Table** **1**, the gate‐connected MoS_2_ ATS‐FET can not only operate in the infrared region, but also exhibit a significantly high photo‐to‐dark current ratio, responsivity, and detectivity. These excellent optical performance characteristics of the gate‐connected MoS_2_ ATS‐FETs indicate that the proposed device has significant potential for application in optoelectronics.

**Table 1:** Performance comparison of gate‐connected MoS_2_ ATS‐FET and recently reported advanced photodetectors

**Device**	**Wavelength [nm]**	***I*_photo_/*I*_dark_**	**Responsivity [A W^−1^]**	**Detectivity [cm Hz^0.5^ W^−1^]**	**Ref**
Gate‐connected					
MoS_2_ ATS‐FET	1550	2 × 10^5^	2.11	2.7 × 10^12^	This work
Multilayer MoS_2_	405	10^3^	3.7		^[^ ^45^ ^]^
Au‐MoS_2_	466	10^5^	20	6 × 10^10^	^[^ ^48^ ^]^
Ta_2_PdS_6_	633		1.4 × 10^6^	7.1 × 10^10^	^[^ ^49^ ^]^
Hybrid‐layered OPT	365	2.9 × 10^6^	8.6 × 10^3^	3.4 × 10^14^	^[^ ^50^ ^]^
Pb—Sn perovskites/InGaZnO	900	50	4.5 × 10^−2^	2.2 × 10^10^	^[^ ^51^ ^]^
Amorphous MoS_2_	473–2712		4.7 × 10^−2^	1.2 × 10^7^	^[^ ^52^ ^]^
MoS_2.15_	445–9536		2.2 × 10^−2^		^[^ ^53^ ^]^
CdTe‐MoS_2_	200–1700	3 × 10^4^	3.7 × 10^−2^	6.1 × 10^10^	^[^ ^54^ ^]^
HgTe quantum dot PT	≈1500–2500	10	>1	>10^11^	^[^ ^55^ ^]^
Si nanomembrane	1550	10^2^	7 × 10^−3^		^[^ ^56^ ^]^
GeSn/Ge	1550	2.3 × 10^2^	99	3 × 10^11^	^[^ ^57^ ^]^
GeSn heterojunction	1550		6.8 × 10^−1^		^[^ ^58^ ^]^

## 3 Conclusion

We developed a gate‐connected MoS_2_ ATS‐FET using a HfO*_x_*:N‐based TS device to overcome the *SS* limit in 2D channel‐based transistors. The proposed ATS‐FET has an extremely low *SS* of 11.1 eV/decade with a range of abrupt current transitions close to 10^4^. The operation mechanism was successfully investigated based on electrical characteristics and circuits, and the main factor determining *V*_TH_ and *SS* is the formation and dissolution of the Ag filament in the TS device. In addition, because the TS device used to obtain a low *SS* is connected to the gate and does not lower the drive current, the ATS‐FET has a high on‐state drive current of ≈10^−6^ A and a high on‐off ratio of ≈10^6^. Moreover, the gate‐connected MoS_2_ ATS‐FET can be used as an infrared detectable phototransistor owing to the detection of infrared light through a Ge gate electrode, which has a narrow bandgap. When the infrared light is incident onto the device, the threshold voltage is shifted from −2.6 to −2.95 V, while maintaining a low *SS*. Owing to the shifting of the threshold voltage and the maintained low *SS*, the proposed device achieves significantly high performance as an optical device, featuring characteristics such as an ultrahigh light‐to‐dark current ratio (4.7 × 10^4^) and high‐selective detectivity (2.7 × 10^12^ cm Hz^0.5^ W^−1^). Owing to these advantages, gate‐connected MoS_2_ ATS‐FETs are considered promising candidates for next‐generation 2D channel‐based electrical and optical devices that require high switching speed and low power consumption.

## 4 Experimental Section

##### Characterization of MoS_2_ Flakes

To confirm the existence and measure the thickness of MoS_2_ flakes, Raman Fourier transform infrared spectroscopy (LabRam ARAMIS IR2, Horiba Jobin Yvon) and AFM (XE‐100, Park systems) were performed. The Raman spectroscopy was performed with an excitation wavelength of 532 nm and a spatial resolution of 1 µm. AFM was performed with lateral and vertical resolutions of 2–3 and 0.1 Å, respectively.

##### Measurement of Electrical and Optical Characteristics

The electrical properties of the fabricated devices were measured using a Keithley 4200‐SCS with laser irradiation at 1550 nm wavelength and without laser irradiation.

## Conflict of Interest

The authors declare no conflict of interest.

## Supporting information

Supporting Information

## Data Availability

Research data are not shared.
