# Supplementary material for: Steep‐Slope Gate‐Connected Atomic Threshold Switching Field‐Effect Transistor with MoS2 Channel and Its Application to Infrared Detectable Phototransistors
Source: Adv Sci (Weinh). 2021 May 3;8(12):2100208. doi: 10.1002/advs.202100208 (PMC8224431; doi:10.1002/advs.202100208)
Supplement: Supplementary file 1 — Supporting Information [file ADVS-8-2100208-s001.pdf]

WILEY-VCH

© 2021 Wiley-VCH GmbH

ADVANCED SCIENCE
Open Access

Supporting Information

for *Adv. Sci.,* DOI: 10.1002/advs.202100208

Steep-slope gate-connected atomic threshold switching field-effect transistor with MoS_2 channel and its application to infrared detectable phototransistors

*Seung-Geun Kim, Seung-Hwan Kim, Gwang-Sik Kim, Hyeok Jeon, Taehyun Kim, and Hyun-Yong Yu**

Supporting Information

Steep-slope gate-connected atomic threshold switching field-effect transistor with MoS_2 channel and its application to infrared detectable phototransistors

*Seung-Geun Kim, Seung-Hwan Kim, Gwang-Sik Kim, Hyeok Jeon, Taehyun Kim, and Hyun-Yong Yu**

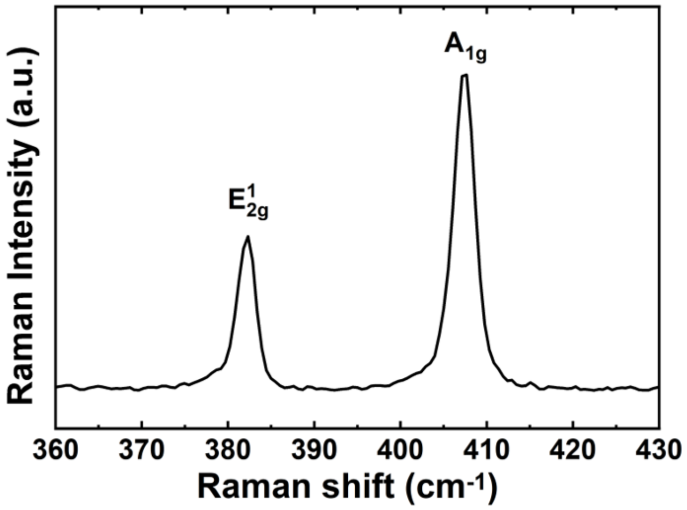

Figure S1. Raman spectra of the exfoliated MoS_2 flake. Two different Raman peaks, E_{2g}^{1} and A_{1g}, were observed near 382.2 and 407.6 cm^{-1} under excitation by a 532 nm line, respectively. The MoS_2 flake used in this work had multi-layers (>5 layers) for comparison with a previous report.

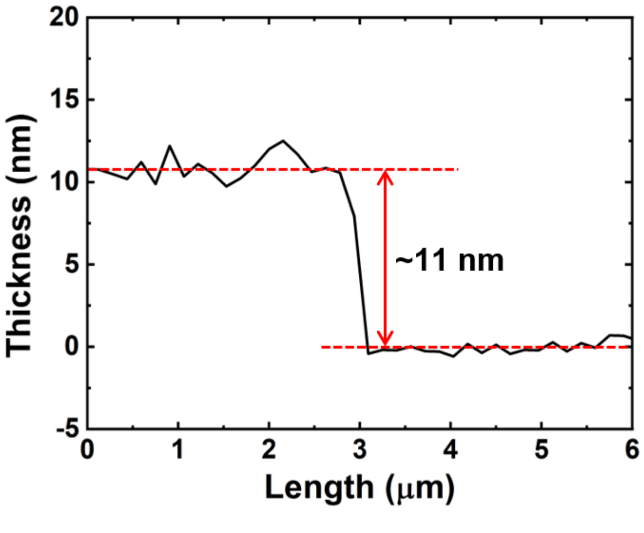

Figure S2. The height profile of the MoS_2 flake. Figure S2 shows height profile of the MoS_2 flake used in this work. The thickness of the MoS_2 flake was measured as approximately 11 nm, which corresponds to approximately 15 layers of MoS_2.

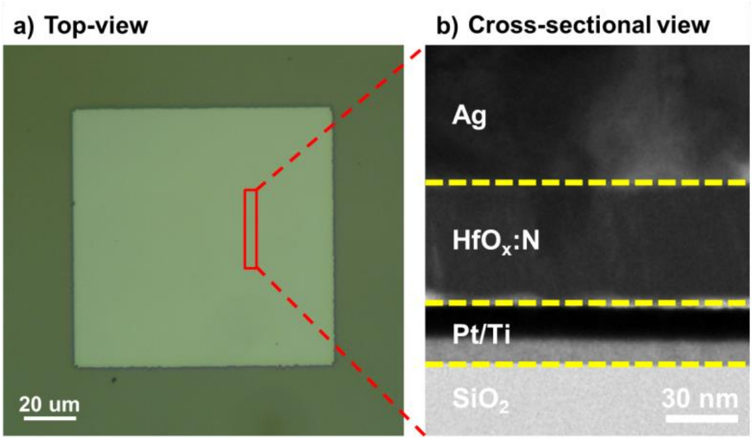

Figure S3. Device image of HfO_x:N-based TS device. a) Top-view optical microscope image, and b) cross-sectional view TEM image.

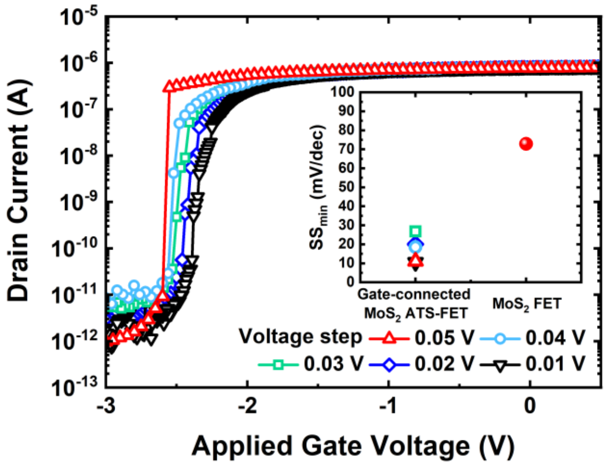

Figure S4. I_D-V_G characteristics of the gate-connected MoS_2 ATS-FET with various V_G steps. The inset shows minimum SS (SS_{min}) values according to the measurement steps.

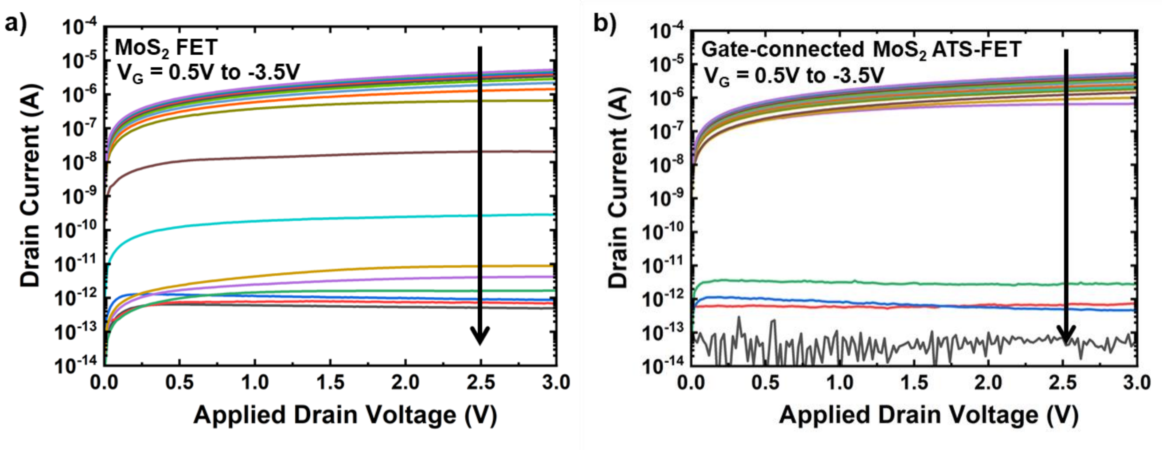

Figure S5. I_D-V_D characteristics of a) MoS_2 FET and b) gate-connected MoS_2 ATS-FET. Gate voltage is applied from 0.5 V to -3.5 V with a -0.25 V step and drain voltage is swept from 0 V to 3 V.

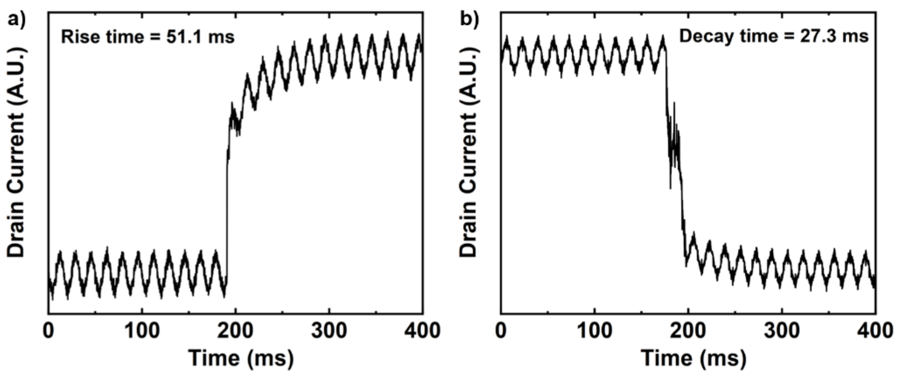

Figure S6. Temporal response of the gate-connected MoS_2 ATS-FET: a) Rising and b) decaying characteristics. The normalized drain current is plotted as a function of measurement time durations and the rise and decay times can be extracted between 10% and 90% of the increasing and decreasing drain current.

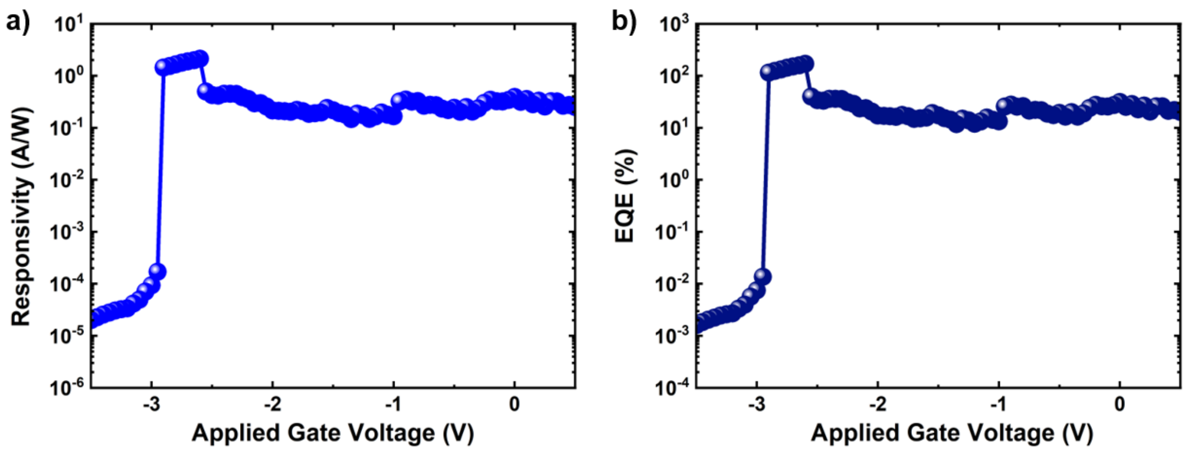

Figure S7. The additory calculated optical properties of the gate-connected MoS_2 ATS-FET related to I_{photo}. a) The responsivity ($R = I_{photo}{P_{eff}}^{-1}$), and b) external quantum efficiency ($\mathrm{EQE} = hcR\lambda^{-1}e^{-1}$) as a function of V_G.

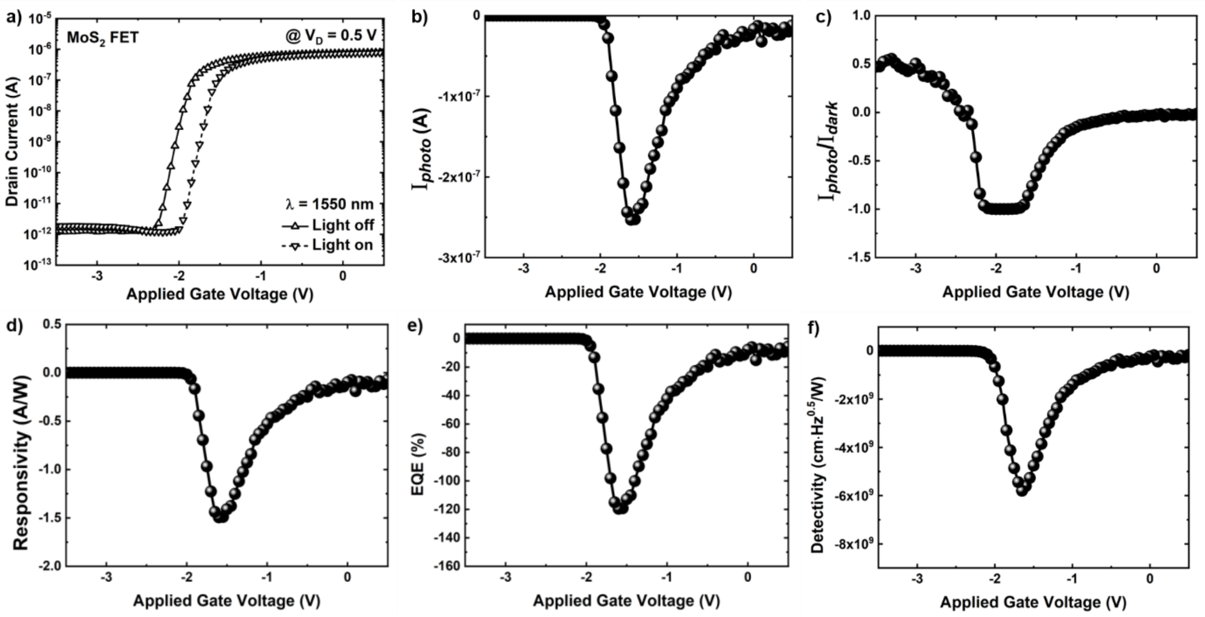

Figure S8. The optical characteristics of the conventional MoS_2 FET with Ge gate electrode. a) I_D–V_G characteristics with and without 1550 nm infrared light, b) the photocurrent, c) the photo to dark current ratio, d) the responsivity, e) the external quantum efficiency, and f) the detectivity.

Table S1. Performance Comparison of Optoelectronic Parameters in Conventional MoS_2 FET and Gate-Connected MoS_2 ATS-FET.

Device	SS [mV/decade]	ΔV_{TH} [V]	I_{photo} [A]	Responsivity [A/W]	EQE [%]	Detectivity [$cmHz^{0.5}/W$]
Conventional MoS_2 FET	73.8	0.29	-2.53×10^{-7}	-1.49	-119.54	-5.80×10^{9}
Gate-connected MoS_2 ATS-FET	11.1	-0.35	3.58×10^{-7}	2.11	169.00	2.71×10^{12}
